# Trends in Cesarean Section Rates in Trinidad and Tobago, 2018-2025: A National Surveillance Study

**DOI:** 10.7759/cureus.104544

**Published:** 2026-03-02

**Authors:** Zada C Mohammed, Adesh Sirjusingh

**Affiliations:** 1 Directorate of Women's Health, Ministry of Health, Port of Spain, TTO

**Keywords:** cesarean section (cs), obstetric care, private healthcare sector, public health services, public health surveillance, retrospective studies, the caribbean region, trinidad and tobago, west indies

## Abstract

Introduction

Cesarean section (CS) rates in Trinidad and Tobago have historically been underdocumented. This study sought to present more recent national data on the CS rate in Trinidad and Tobago, explore and analyse the observed trends, and project future trajectories of CS rates to 2030.

Methods

This was a retrospective descriptive study utilizing aggregated, de-identified surveillance data on births and CS from 2018 to 2025. Chi-square tests of independence were performed to assess the association between the mode of delivery and the health sector (public or private) annually. Linear regression was used to assess time trends in CS rates and project CS rates for 2030.

Results

Analysis of 110,347 births revealed that the national CS rate increased from 29.6% (4,979/16,849) in 2018 to 38.4% (4,137/10,779) in 2025. The private sector CS rate was consistently higher than the public sector rate, increasing from 51.9% (910/1,753) in 2018 to 61.1% (580/949) in 2025, while the public sector rate increased from 27.0% (4,069/15,096) in 2018 to 36.2% (3,557/9,830) in 2025. The differences between public and private sector CS rates were statistically significant for all years (*χ^2^* range: 227.46-469.90; p < 0.001). Simple linear regression revealed that nationally, CS rates increased significantly across all levels (*t*-statistic range: 9.01-9.56; p < 0.001). The average annual increase in CS rates was 1.13 percentage points (95% CI: 0.84-1.42) nationally, 1.16 percentage points (95% CI: 0.86-1.45) in the public sector, and 1.33 percentage points (95% CI: 0.97-1.69) in the private sector. Linear trend projections forecast an increasing national CS rate of 43.5% (95% PI: 40.3%-46.6%) by 2030, 41.3% (95% PI: 38.0%-44.6%) in the public sector, and 68.3% (95% PI: 64.3%-72.2%) in the private sector.

Conclusion

The CS rate in Trinidad and Tobago is high and far exceeds the levels associated with reductions in maternal and neonatal mortality. The significant disparity between the public and private sector CS rates is suggestive of non-clinical factors potentially influencing the mode of delivery. Although limited by the use of aggregated data, the findings are suggestive of an urgent need to review indications for CS to ensure that the procedure is used appropriately and equitably.

## Introduction

Childbirth is a fundamental human experience, and the mode of delivery has significant implications for maternal and neonatal short- and long-term health. Cesarean section (CS) is a critical surgical intervention for managing obstetric complications, proven to save lives when medically indicated. However, when compared with a vaginal delivery, CS has a higher risk of surgical complications, wound infection, hospital stay, uterine rupture, and morbidly-adherent placenta in future pregnancy, emergency hysterectomy, maternal death, and anesthesia-related risks [1,2]. There is also a higher reported risk of neonatal morbidity and mortality [3], but perinatal outcomes may improve as reported from Trinidad and Tobago [4]. 

In a 2014 systematic review, the World Health Organization (WHO) found that CS rates higher than 10%, and even up to 30%, were not associated with reductions in maternal and neonatal mortality [5]. In April 2015, the WHO issued a statement on CS rates, recommending that every effort should be made to provide CS to women who need it, rather than trying to achieve a specific target for the CS rate [5].

Globally, CS rates have surged over the past three decades, with the average CS rate increasing 19 percentage points from 1990 to 2018 [6], and CS becoming the most frequent major abdominal surgery [7]. Based on data up to 2018, the global CS rate stood at 21.1%, with the highest rate (42.8%) in Latin America and the Caribbean (LAC). Projections indicate that by 2030, the global CS rate will rise to 28.5%, with the LAC region reaching 54.3% [6].

Trinidad and Tobago is a high-income developing twin-island nation in the Caribbean with a 2025 mid-year population estimate of 1,367,764 [8]. The healthcare system consists of both the public and private sectors. In the public health sector, there are six maternity units. In public hospitals, health care, including CS, is free at the point of service delivery. In private hospitals, health care is self-funded and/or covered by private health insurance. In Trinidad and Tobago, approximately 90% of births are in the public sector [9]. 

Historically, there has been a lack of recent, representative, and consistent national data on CS in Trinidad and Tobago. Estimates of CS rates from previous studies include 6.6% in one teaching hospital in 1986 [10], 7.4% based on a seven-year retrospective study in 2001 [11], and 14.4% in a 2010 survey of 368 women who were attending antenatal and postnatal clinics in north Trinidad [12]. Estimates of national CS rates are available from the Multiple Indicator Cluster Survey (MICS), which reports the percentage of women aged 15-49 years whose most recent live birth in the last two years was delivered by cesarean section. Data from the 2011 round of the MICS indicated a CS rate of 22.1% [13], and the 2022 round of the MICS found that this increased to 29.6% [14].

The more recent estimates surpass 10%, above which there is no association with reductions in maternal and neonatal mortality. It therefore appears that Trinidad and Tobago is mirroring the global trend of CS rates, potentially extending beyond medical necessity. This raises concerns about the potential over-medicalization of birth, unnecessary healthcare expenditure, and the long-term health of the Trinidadian population.

Therefore, the objectives of this study were to present recent national data on the overall CS rate in Trinidad and Tobago; explore and analyse the observed trends from 2018 to 2025, specifically comparing the public and private health sectors; and project future trajectories of national and sector-specific CS rates to 2030.

## Materials and methods

Study design and setting

This study was a retrospective descriptive analysis of CS rates in Trinidad and Tobago. The study population included all births in the public and private health sectors from January 1, 2018, to December 31, 2025.

Data source and collection

Data for this study were obtained from the Trinidad and Tobago Maternal, Perinatal, and Adolescent Health Surveillance System (TT MPAHSS), a national electronic passive surveillance system created in 2017 and managed by the Directorate of Women’s Health in the Ministry of Health, Trinidad and Tobago [15]. The system collects monthly data on key maternal and neonatal morbidity and mortality indicators, including births and CS, from all six public and 11 private maternity units in Trinidad and Tobago. Internal Ministry of Health approvals were obtained to create, implement, revise, and monitor the system. A link to a de-identified demonstration version of the TT MPAHSS, illustrating the variable structure and interface, is provided in the Appendices. 

In terms of data validation and quality control, submissions are reviewed for completeness, timeliness, and inconsistencies (e.g., accuracy of calculated values, comparison to past submissions, etc.). However, validation does not involve cross-checking aggregated data against facility clinical records. Data security is maintained at all times, with the dataset stored on secure, password-protected systems and access restricted to authorized personnel to ensure confidentiality.

For this analysis, data on the annual number of births and number of CS, disaggregated by public and private health sectors, were extracted from the TT MPAHSS database. There was 100% reporting from all 17 health facilities during the study period. As the birth data utilized in this study were derived from a surveillance system rather than the national civil registry, it should be noted that these figures represent operational datasets and are considered provisional pending the release of finalized vital statistics by the relevant national authorities.

Ethical considerations

This study utilized de-identified aggregated surveillance data obtained from the TT MPAHSS, which does not include individual patient data and clinical details. As such, the study was exempt from formal ethics review. Approval to collect this surveillance data was obtained from the Ministry of Health Executive (File Number: He: 11/1/100 Vol. 1 Folio (2), Approval Date: August 25, 2017).

Variables and definitions

The primary outcome variable for this study was the CS rate, defined as the percentage of deliveries by CS among all deliveries, irrespective of vital status at birth (live birth and stillbirth), in a health facility during a specified reference period [16]. The CS rate was calculated as the number of deliveries by CS divided by the total number of deliveries, multiplied by 100. The other variables considered were year, which was used for trend analysis, and health sector, a categorical variable with two levels - public or private. 

Statistical analysis

All statistical analyses were performed using Stata 13 (StataCorp, College Station, USA). Statistical significance was set at p < 0.05. Annual CS rates were calculated at the national level and for the public and private sectors for each year of the study period. Trends in the CS rates over time were visualized with a line graph.

Chi-square tests of independence were conducted for each year during the study period to assess the association between the mode of delivery and health sector (public or private) using the numbers of CS and vaginal deliveries in each sector. The null hypothesis was that there was no association between the mode of delivery and the health sector.

To examine the trends in CS rates over the study period, time-series analysis was performed. For the national level, and the public and private sectors, simple linear regression was used to quantify the average annual change in CS rates and assess the statistical significance of the trend. Diagnostic testing was conducted for all three models (national, public, and private) to ensure statistical validity. The Shapiro-Wilk test confirmed the normality of residuals (p > 0.05 for all models), and the Breusch-Pagan test confirmed homoscedasticity (p > 0.05 for all models). The Durbin-Watson test indicated acceptable independence of errors for the national and public sector models (d = 2.46 and 2.31, respectively), while negative autocorrelation was observed in the private sector model (d = 3.31). Despite this autocorrelation, which was deemed to likely be an artifact of the small sample size (n = 8), the overall robust adherence to normality and homoscedasticity supported the use of linear models for forecasting. Projections for 2030 were calculated with 95% prediction intervals (PI) to quantify uncertainty based on the *t*-distribution to account for the small sample size.

## Results

From 2018 to 2025, there were 110,347 births in health facilities - 99,903 (90.5%) in the public sector and 10,444 (9.5%) in the private sector. The overall national CS rate for the eight years was 33.5% (36,959/110,347). The CS rate in the public sector was 31.1% (31,071/99,903) compared to 56.4% (5,888/10,444) in the private sector, indicating that for the eight years, the CS rate in the private sector was approximately 1.8 times that of the public sector CS rate. 

Table 1 shows that while the number of births decreased over the study period, the CS rate increased. The Chi-square analyses demonstrated a statistically significant association between the mode of delivery and the health sector for every year of the study (*χ^2^* range: 227.46-469.90; df = 1; p < 0.001).

**Table 1:** National and sector-specific cesarean section (CS) rates and birth statistics. 1. The birth data utilized in this study were derived from a surveillance system and not the national civil registry. These figures represent operational datasets and are considered provisional pending the release of finalized vital statistics by the relevant national authorities. 2. The percentage of CS deliveries represents the CS rate.

Year	National			Public Sector			Private Sector			χ2 results (mode of delivery vs health sector)	
	Total births	CS deliveries (%)	Vaginal deliveries (%)	Total births	CS deliveries (%)	Vaginal deliveries (%)	Total births	CS deliveries (%)	Vaginal deliveries (%)	Test statistic	p-value
2018	16,849	4,979 (29.6)	11,870 (70.4)	15,096	4,069 (27.0)	11,027 (73.0)	1,753	910 (51.9)	843 (48.1)	469.90	p < 0.001
2019	15,691	4,981 (31.7)	10,710 (68.3)	14,215	4,179 (29.4)	10,036 (70.6)	1,476	802 (54.3)	674 (45.7)	383.78	p < 0.001
2020	15,160	4,811 (31.7)	10,349 (68.3)	13,763	4,043 (29.4)	9,720 (70.6)	1,397	768 (55.0)	629 (45.0)	383.64	p < 0.001
2021	14,839	5,054 (34.1)	9,785 (65.9)	13,383	4,253 (31.8)	9,130 (68.2)	1,456	801 (55.0)	655 (45.0)	315.64	p < 0.001
2022	13,250	4,584 (34.6)	8,666 (65.4)	11,928	3,803 (31.9)	8,125 (68.1)	1,322	781 (59.1)	541 (40.9)	388.96	p < 0.001
2023	12,891	4,412 (34.2)	8,479 (65.8)	11,799	3,775 (32.0)	8,024 (68.0)	1,092	637 (58.3)	455 (41.7)	308.02	p < 0.001
2024	10,888	4,001 (36.7)	6,887 (63.3)	9,889	3,392 (34.3)	6,497 (65.7)	999	609 (61.0)	390 (39.0)	277.46	p < 0.001
2025	10,779	4,137 (38.4)	6,642 (61.6)	9,830	3,557 (36.2)	6,273 (63.8)	949	580 (61.1)	369 (38.9)	227.47	p < 0.001

**Figure 1:**
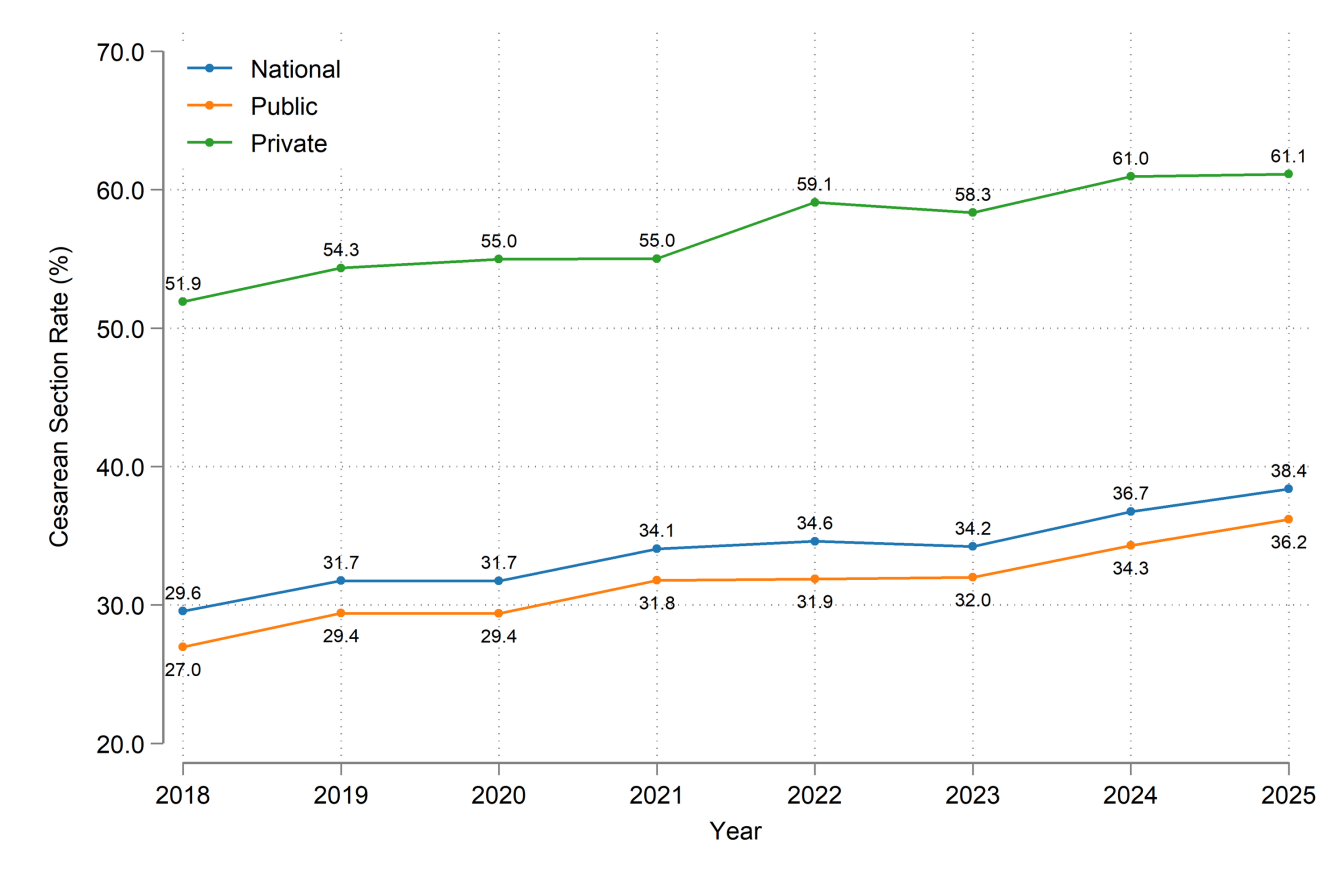
Cesarean section rates in Trinidad and Tobago, 2018 - 2025. For the exact frequencies, please refer to Table 1.

Figure 1 shows the increasing trend in CS rates nationally as well as in the public and private sectors. Private sector CS rates were consistently higher than the public sector CS rates. Notably, the absolute difference in CS rates between the two sectors was relatively stable (range: 23.3%-27.2%) throughout the eight years.

Table 2 shows the linear regression models obtained from the time series analyses and the prediction values for CS rates in 2030 based on those models. Linear regression confirmed statistically significant increases in CS rates nationally (*t* = 9.56, p < 0.001), and in the public (*t* = 9.48, p < 0.001) and private sectors (*t* = 9.01, p < 0.001). Further, the R² values indicate a strong effect size, demonstrating that the passage of time alone explains over 93% of the variance in the increasing CS rates across all three models. From the regression model, the national CS rate increased by an average of 1.13 percentage points per year, every year during the period 2018 to 2025. Comparing the public and private sectors, the increase in CS rates was more pronounced in the private sector, which saw an average annual rise of 1.33 percentage points per year, compared to 1.16 percentage points per year in the public sector. Based on these linear trends, the models predicted a continued increase across all sectors by 2030, with the national and public sector CS rates predicted to increase to over 40%, and the private sector's rate predicted to exceed 65%.

**Table 2:** Linear regression and projected 2030 cesarean section (CS) rates. *Note: Coefficients represent the average annual increase in percentage points.

Level	Linear regression				2030 CS rate projection (95% PI)
	Average annual increase (95% CI)*	R^2^	*t*-statistic	p-value	
National	1.13 (0.84-1.42)	0.9384	9.56	< 0.001	43.5% (40.3%-46.6%)
Public Sector	1.16 (0.86-1.45)	0.9374	9.48	< 0.001	41.3% (38.0%-44.6%)
Private Sector	1.33 (0.97-1.69)	0.9312	9.01	< 0.001	68.3% (64.3%-72.2%)

## Discussion

At the population level, the CS rate reflects the accessibility to and the use of CS, and can help policymakers monitor progress in maternal and infant health as well as resource utilization [5]. When used for medically indicated reasons, CS is an effective intervention in reducing mortality. However, excessive use exposes women to unnecessary surgical risks.

This study reveals a statistically significant increase in CS rates in Trinidad and Tobago from 2018 to 2025. National CS rates, and CS rates in the public and private sectors, have risen far beyond 10%, above which there is no association with reductions in maternal and newborn mortality [5]. The national, public sector, and private sector CS rates are higher than the 2018 global CS rate estimate (21.1%), but lower than the 2018 estimate for LAC (42.8%) [6]. The 2030 projected CS rates are also much higher than the 2030 global projection (28.5%) but lower than the 2030 LAC projection (54.3%) [6]. Increasing trends in CS rates have been documented globally [6] and are potentially influenced by a confluence of medical, socio-cultural, and systemic factors.

Medical factors

Several medical and obstetric conditions are established indications for CS. These include previous CS (one of the leading indications for repeat CS); fetal distress and suspected macrosomia; and maternal conditions such as pre-eclampsia, gestational hypertension, and gestational diabetes mellitus [17]. 

In its 2021 review of CS rates, the Scottish government found that the proportion of births delivered by CS had increased to a greater extent for older mothers, and the proportion of overweight or obese mothers giving birth in Scotland had also increased. A combination of higher maternal age and increasing levels of obesity and chronic disease, such as diabetes, meant that more pregnancies were medically complex, resulting in a higher risk during pregnancy, labour, and birth, which could be associated with a higher rate of CS [18]. Additionally, a 2021 study by Colaci found that in LAC, cesarean delivery was associated with maternal age [19]. 

Increasing high-risk pregnancies due to increasing maternal age and increasing prevalence of obesity and chronic disease could be possible explanations for increasing CS rates in Trinidad and Tobago. Data from the 2011 and 2022 rounds of the MICS are consistent with a demographic shift in Trinidad and Tobago toward older mothers. Between 2011 and 2022, fertility rates among women in their 20s dropped by over 20%, whereas the fertility rates among women in their 30s experienced a decline of approximately 10%. Further, the fertility rate among women ages 40-44 years was unchanged (12 live births per 1000 women). As such, the more pronounced reduction in younger women is consistent with a greater proportion of the obstetric population now being composed of women over 30 years of age, potentially raising the average risk profile of this group [13-14]. 

With respect to chronic diseases, the 2011 WHO STEPwise approach to noncommunicable disease (NCD) risk factor surveillance (STEPS) survey revealed that 59.0% of women ages 15-64 years in Trinidad and Tobago were overweight or obese, 23.1% had raised blood pressure or were currently on antihypertensive medication, and 21.2% had raised blood glucose or were currently on medication for raised blood glucose [20]. In the 2024 STEPS, these figures increased to 67.2% and 24.9% for overweight/obese and raised blood pressure, respectively, but decreased to 17.3% for raised blood glucose [21]. This is suggestive of a high prevalence of NCD risk factors among women in Trinidad and Tobago, which potentially increases the likelihood of more high-risk pregnancies and delivery by CS.

Non-medical factors

Beyond medical necessity, non-clinical factors potentially influence delivery modes. In the LAC region, cesarean deliveries have been found to be associated with maternal education, household income or wealth, and urban residency [19]. 

Maternal request is a growing phenomenon common among older, more educated, and higher-income women in the private sector because of fear of labour pain, perceived safety for the baby, and convenience (scheduling). Additionally, medico-legal concerns, where the fear of malpractice lawsuits in cases of adverse perinatal outcomes from vaginal delivery, create a defensive practice style that favours surgical intervention. Provider practices, e.g., convenience for the obstetrician (scheduling deliveries) and a gradual erosion of skills in managing complex vaginal deliveries (e.g., operative vaginal delivery with forceps/vacuum) are also contributing factors [22-23]. Examples of systemic factors include fragmented antenatal care, lack of widespread access to continuous labour support (e.g., doulas), and insufficient institutional support for evidence-based practices like vaginal birth after cesarean section (VBAC) and trial of labour after cesarean (TOLAC) [22-23].

These non-medical factors are potentially influencing the increasing CS rates in Trinidad and Tobago, as evidenced by 2022 MICS data, which revealed that nearly half (14.1% of 29.6%) of women who delivered by CS decided on the procedure before the onset of labour pains [14]. This is suggestive of a substantial portion of CS in Trinidad and Tobago being potentially planned or elective procedures, a trend that aligns with recent findings from other developing contexts, such as a tertiary care centre in India, which reported an almost doubling of CS deliveries because of maternal request from 2016 (8.77%) to 2021 (15.70%) [24]. In Trinidad and Tobago, decisions regarding the mode of delivery are potentially influenced by a lack of accurate clinical information. While recent local data on maternal health literacy are limited, a 2010 study by Mungrue et al found that the majority of women (61.7%) attending antenatal and postnatal clinics had little to no knowledge on CS to make informed choices when choosing a mode of delivery, and that the main sources of information were from a relative/friend (50%) and mass media (28.5%) [12].

Public and Private Sector Disparities

A statistically significant difference was observed between CS rates in the public and private health sectors. The private sector's rates were consistently above 50% and were approximately 1.8 times that of the public sector. This is similar to a study conducted by Boerma et al, which found that globally, CS rates were about 1.6 times higher in private facilities compared to public facilities [25]. Higher CS rates in private facilities have been documented across country contexts, for example, the Dominican Republic [19] and Australia [26]. 

One commonly identified factor contributing to this disparity relates to the differing financial and service delivery models between sectors. As evidenced by Hoxha et al in their systematic review and meta-analysis, CS was more likely to be performed in for-profit hospitals compared to non-profit hospitals, regardless of women’s risk and other contextual factors [27]. This is suggestive of structural institutional factors potentially influencing delivery trends in Trinidad and Tobago, particularly as healthcare is free at the point of service delivery in the public sector, while the private sector is self-funded. Other factors potentially influencing higher CS rates in the private sector include convenience, choice of physician, continuity of care, and privacy while giving birth [28]. Of note, a 2018 study by Rivo et al found that in Argentina, private sector providers were more willing to perform a cesarean section on maternal request as compared to providers in the public sector [29].

The relatively stable difference in CS rates between the two sectors for each year of the study period is consistent with the premise that the postulated medical and non-medical factors described above - a high-risk maternal profile, an increasingly litigious environment prompting defensive medicine, and a broader cultural shift towards surgical delivery - are potentially influencing both sectors similarly.

Future projections and policy implications

The linear regression models project that by 2030, the national CS rate would exceed 40%, and the private sector's rate is forecasted to surpass 65%. As such, if current trends persist, the CS is likely to account for a greater proportion of deliveries, especially in the private sector. As shown in Table 1, Trinidad and Tobago is currently experiencing a declining birth rate. While this is suggestive of a reduced burden on obstetric services, with a rising CS rate, it means that a greater proportion of expectant mothers are more likely to undergo a major surgical procedure.

This trend, when viewed alongside the high prevalence of obesity and NCD risk factors identified in the STEPS surveys, necessitates a shift in national health policy towards integrating high-risk maternal screening more aggressively into early antenatal care. This also means more healthcare resources, e.g., use of operating theatres and surgical staff would be required. Strategic workforce planning and the development of standardized clinical audit systems are essential to ensure that surgical capacity meets this projected demand without compromising clinical safety.

Strengths and limitations

This study presents, for the first time, comprehensive, consistent, and recent national data on CS in Trinidad and Tobago. The primary strength of this study is the use of national surveillance data, which all maternity units - public and private - are a part of. In Trinidad and Tobago, more than 99% of births occur in a health facility [14]. As such, the data analysed in this study are representative. Further, the availability of eight years of data allows for the establishment of a sustained trend, and not just a cross-sectional snapshot.

This study has several limitations. First, the use of aggregated, self-reported data from a passive surveillance system introduced potential reporting bias and did not allow for analysis of individual patient clinical details such as maternal age, parity, and specific indications, including primary versus repeat CS. Further, the absence of national-level Robson classification data restricted the ability to make comparisons between sectors. The population-level approach utilized in the study subjected the analysis to ecological fallacy, meaning that the relationships between CS rates and the potentially associated medical and non-medical factors remain associative rather than causal. Additionally, the short time-series (n = 8 years) limited advanced modelling and introduced statistical fragility, such as the negative autocorrelation observed in the private sector. The 2030 linear projections should be interpreted with caution, as any future changes in policy or practice could also change these estimates. Finally, the findings of this study may not be generalizable to settings where public healthcare is not free at the point of care or with different public-private delivery ratios. Future research must utilize individual-level data to identify the demographic and clinical indications associated with increasing CS rates.

Recommendations

While increasing CS rates in Trinidad and Tobago may be attributed to legitimate factors such as older maternal age and higher rates of NCDs, the scale of the increase is suggestive of a significant proportion of CS being potentially influenced by factors beyond strict medical indication. Reversing this trend, therefore, requires a coordinated multi-stakeholder approach.

Short-term facility-level recommendations

In the short term, health facilities should conduct comprehensive clinical audits of all deliveries. Utilizing the Robson classification system to document the indication for every CS will provide the granular data required to identify primary drivers and pinpoint areas for targeted intervention. There needs to be more focus on providing continuous support for women during childbirth and optimizing the management of labour to reduce the need for surgical delivery. This includes actively promoting and supporting safe Assisted Vaginal Birth (AVB) for women requiring assistance in the second stage of labour. AVB, when successful, leads to lower maternal morbidity compared to a cesarean delivery at full dilation, and women are far more likely to have a spontaneous vaginal birth in their next pregnancy [30]. Healthcare providers should be trained in the management of labour and instrumental delivery. Qualitative studies, such as interviews and focus groups with patients and providers, can also be conducted to understand how the social determinants of health and other non-clinical factors potentially influence CS rates.

Long-term national recommendations

Over the long term, national strategies should focus on the development, implementation, and enforcement of robust, evidence-based clinical guidelines for CS and the appropriate use of TOLAC/VBAC. These guidelines can also serve as a structured legal defence for practitioners who adhere to them when opting for vaginal delivery, helping to mitigate defensive medicine. Midwifery-led care, the integration of midwives and doulas into the care model to provide continuous support during labour, should be promoted as it has been shown to reduce the likelihood of CS [18]. Finally, a sustained public health campaign should be launched to educate women and their families on the benefits and risks of both vaginal and cesarean delivery, empowering informed decision-making and improving maternal health literacy across the population.

## Conclusions

This study presents current data on CS rates in Trinidad and Tobago. The high and rising CS rate, with disproportionately higher rates in the private health sector, is a complex public health issue and is potentially influenced by factors beyond medical necessity. While CS remains a vital life-saving procedure, potential overuse can lead to an increased risk of maternal complications, including hemorrhage, infection, and surgical injury. This is suggestive of a departure from evidence-based obstetric care and places a considerable strain on the country’s healthcare resources. Addressing this challenge is imperative to safeguard maternal and neonatal health, ensure the equitable and efficient use of resources, and preserve the normal physiological process of childbirth where possible. A concerted effort from policymakers, healthcare providers, and the public is needed to cultivate a culture that values and supports safe vaginal birth while reserving CS for when it is truly needed.

## Appendices

Trinidad and Tobago Maternal, Perinatal, and Adolescent Health Surveillance System (TT MPAHSS)

The TT MPAHSS databases are not publicly available. However, the following link provides access to a demonstration version of the TT MPAHSS. This version mirrors the variable fields and data entry structure used in the actual surveillance system but contains no real patient data. 

URL: https://docs.google.com/spreadsheets/d/1WybVIQWBUuQ_AYYrT-IIamj8EfnJvAeJnb1ye7WgIho/edit?usp=sharing.
